# Surgical Management of Pterygium Invading the Corneal Flap Following Laser In Situ Keratomileusis: A Case Series

**DOI:** 10.7759/cureus.95968

**Published:** 2025-11-02

**Authors:** Aki Yoshida, Ami Igarashi, Toshiki Shimizu, Satoru Yamagami, Takahiko Hayashi

**Affiliations:** 1 Ophthalmology, Nihon University School of Medicine, Tokyo, JPN

**Keywords:** case series, conjunctival flap grafting, laser in situ keratomileusis, lasik, pterygium

## Abstract

Pterygium is a benign fibrovascular growth that may invade the cornea and cause irregular astigmatism or visual disturbance. Surgical excision with conjunctival autografting is the standard treatment to prevent recurrence. However, the management of pterygium extending into the corneal flap after laser in situ keratomileusis (LASIK) is rarely reported and presents unique challenges due to the risk of flap-related complications. We report four cases of pterygium excision with conjunctival flap grafting for pterygium invasion of the corneal flap following LASIK treatment. This case series included four eyes of four patients (three men and one woman; mean age: 52.8±5.1 years) who received treatment at Nihon University Itabashi Hospital between November 2021 and February 2025. All patients underwent pterygium excision and conjunctival flap transplantation for the pterygium extending beneath the LASIK flap. The pre- and postoperative visual acuity, corneal astigmatism, and higher-order aberrations (HOAs) were measured using anterior segment optical coherence tomography (AS-OCT; CASIA 2, Tomey, Aichi, Japan). The mean follow-up period was 12.48±16.06 months. None of the patients experienced intra- or postoperative complications. The mean corrected logMAR visual acuity remained stable from baseline (-0.15±0.04) to the final visit (-0.15±0.04). The mean corneal astigmatism decreased from 3.33±2.54 D to 1.08±0.32 D (p=0.25). Anterior corneal HOAs (6 mm) improved from 3.21±2.38 μm to 0.99±0.23 μm (p=0.125). Posterior corneal HOAs (6 mm) were largely stable, changing from 0.22±0.11 μm to 0.12±0.02 μm (p=0.11). In conclusion, pterygium excision with conjunctival flap grafting is a safe and effective treatment for pterygia invading the LASIK flap. To prevent flap displacement and other complications, it is crucial to take special care to avoid tension on the LASIK flap during surgery.

## Introduction

Pterygium is a common ocular surface disorder, characterized by the progressive growth of fibrovascular tissue from the conjunctiva to the cornea, with reported prevalence rates ranging from 1.2% to 23.4% [1]. Extension of pterygium lesions to the mid-peripheral cornea or beyond commonly induces significant corneal astigmatism, requiring surgical removal to enhance visual function [2].

Surgical treatment options for pterygium include simple bare sclera excision, which is associated with high recurrence rates [3]; conjunctival autografting using either free or rotated flaps with sutures or fibrin glue, which shows lower recurrence rates [4]; and several adjuvant therapies such as mitomycin C, amniotic membrane transplantation, or lamellar keratoplasty, which have been reported to minimize the risk of recurrence in complex cases such as recurrent or highly vascularized pterygia [5-7].

Although the precise pathophysiological mechanisms underlying the development of pterygium remain unclear, several contributory risk factors have been identified, including chronic exposure to ultraviolet radiation, ocular surface irritation, and trauma [8]. Lopez and Sayegh previously reported that pterygium may also develop following laser in situ keratomileusis (LASIK), possibly due to alterations in ocular surface homeostasis or wound healing dynamics [9].

Despite the increasingly widespread use of LASIK, reports of pterygium formation in post-LASIK eyes remain limited. Furthermore, to the best of our knowledge, no study has yet provided a detailed evaluation of surgical outcomes, recurrence rates, or changes in visual function, including higher-order aberrations (HOAs), in such cases.

The corneal flap created during LASIK lacks suture stabilization and thus may be susceptible to external stress, including trauma or subsequent surgical manipulation [10]. These anatomical considerations underscore the need for caution regarding the safety and efficacy of pterygium excision in post-LASIK eyes, particularly when the lesion encroaches upon or involves the corneal flap.

This study evaluated the surgical outcomes of pterygium excision with conjunctival flap transplantation in post-LASIK patients, further describing the detailed surgical technique and outcomes, with a particular focus on recurrence and postoperative visual quality, including HOAs. This study aims to demonstrate a surgical approach to achieve both functional and cosmetic success in pterygium cases after LASIK.

## Case presentation

Study design and patients

This retrospective study enrolled four eyes of four patients who underwent surgery at Nihon University Itabashi Hospital between November 2021 and February 2025. The inclusion criteria were eyes with a pterygium invading the LASIK flap, treated with pterygium excision and conjunctival flap transplantation.

This study was approved by the Ethical Review Board of the Nihon University School of Medicine (approval number: RK-250909-3) and adhered to the tenets of the Declaration of Helsinki. All patients provided informed consent in the form of opt-out on the website. Those who rejected were excluded.

Examinations

Best-corrected visual acuity (BCVA), corneal astigmatism, and intraocular pressure (IOP) were measured pre- and postoperatively. The decimal values of BCVA were converted to logarithmic values for statistical analysis. Corneal HOAs with a minimum central diameter of 6 mm were evaluated using the CASIA 2 system (SS1000, Tomey, Aichi, Japan).

Corneal HOAs

Zernike coefficients were derived through a standard Zernike polynomial analysis, following established methodologies. Three-dimensional reconstructions of both the anterior and posterior corneal surfaces were generated from corneal elevation data. HOAs were subsequently quantified for 6 mm optical zones using CASIA with the built-in ray-tracing software (version 5.1). The refractive indices used in the analysis were 1.376 and 1.336 for the cornea and aqueous humor, respectively. The HOAs were computed as the root mean square of the third- to eighth-order Zernike coefficients.

Surgical technique

**Figure 1:**
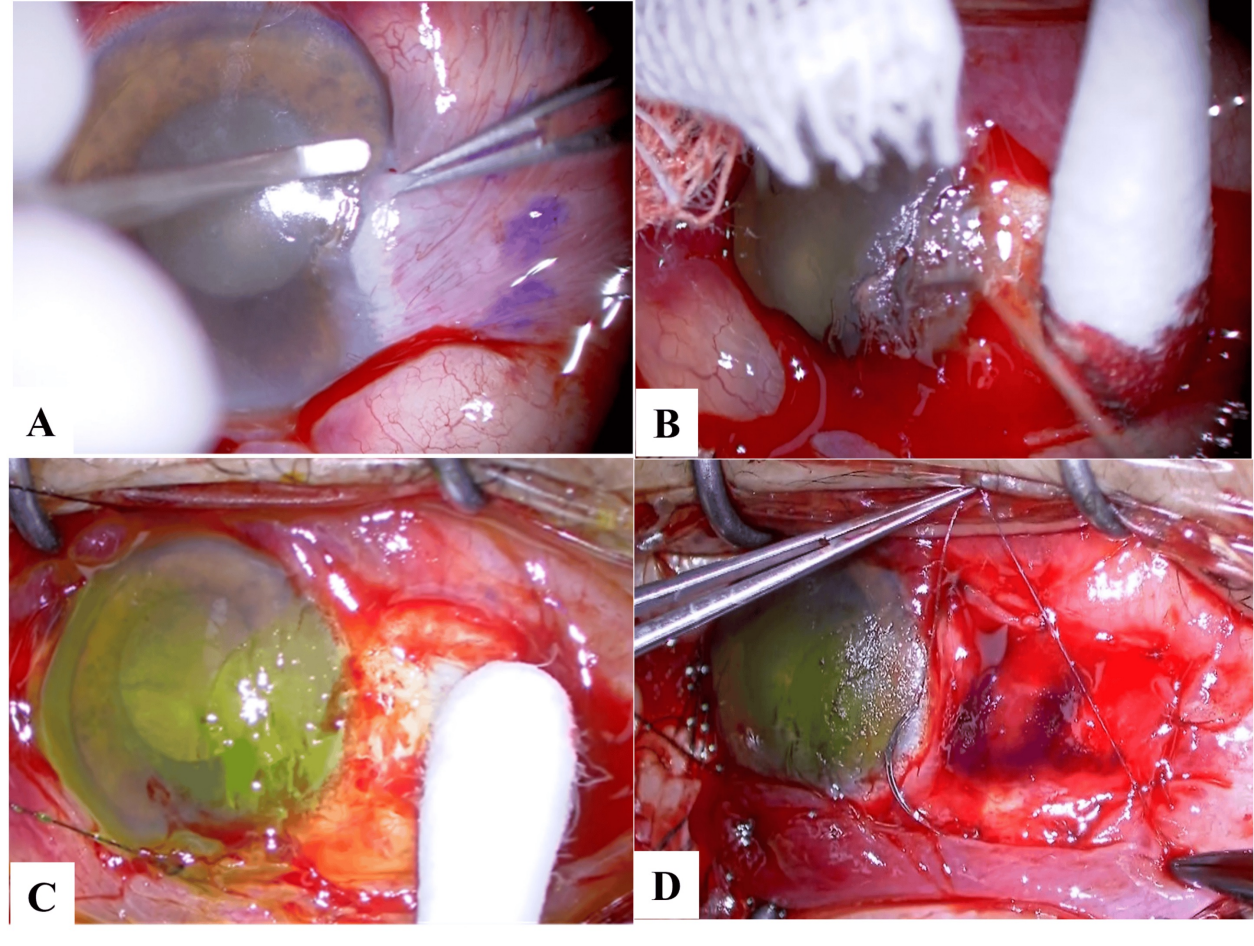
Overview of the surgical technique for the excision of pterygium following LASIK (A) First, subconjunctival anesthesia was administered; subsequently, a blunt DLK spatula was used to dissect the head of the pterygium without injuring the LASIK flap. (B) The anterior corneal surface was carefully smoothed by gentle scraping. (C) The flap integrity was checked in real time, and damage to the LASIK flap was evaluated using fluorescein staining. (D) Finally, the conjunctival flap was transplanted onto the bare sclera. DLK: deep lamellar keratoplasty; LASIK: laser in situ keratomileusis

All procedures were conducted under local anesthesia. To facilitate tissue separation by promoting hydrodissection and ease the dissection of the superficial epithelium from the underlying layers, 2% lidocaine was injected into the conjunctival stroma. The pterygium was excised from the corneal flap using a Maeda-type deep lamellar keratoplasty (DLK) spatula (Inami, Tokyo, Japan), with care taken to avoid applying tension to the LASIK flap. After removing the pterygium, the corneal surface was gently polished using a spatula to ensure a smooth stromal bed. Fluorescein staining (AYUMI Pharmaceutical Corporation, Tokyo, Japan) was then performed intraoperatively to verify the depth and area of tissue excision. The conjunctival flap was then rotated to cover the scleral area. Finally, the graft was sutured to the host area using an 8-0 Vicryl. This procedure is outlined in Figure 1 and Video 1.

**Video 1:** A video of the surgical technique applied to excise the pterygium following LASIK The pterygium tissue was carefully excised from the LASIK flap using a Maeda-type DLK spatula (Inami) to avoid damaging the LASIK flap. After removing the pterygium, the corneal surface was evaluated using fluorescein staining. A conjunctival graft was sutured to the host area using an 8-0 Vicryl. LASIK: laser in situ keratomileusis

**Figure 2:**
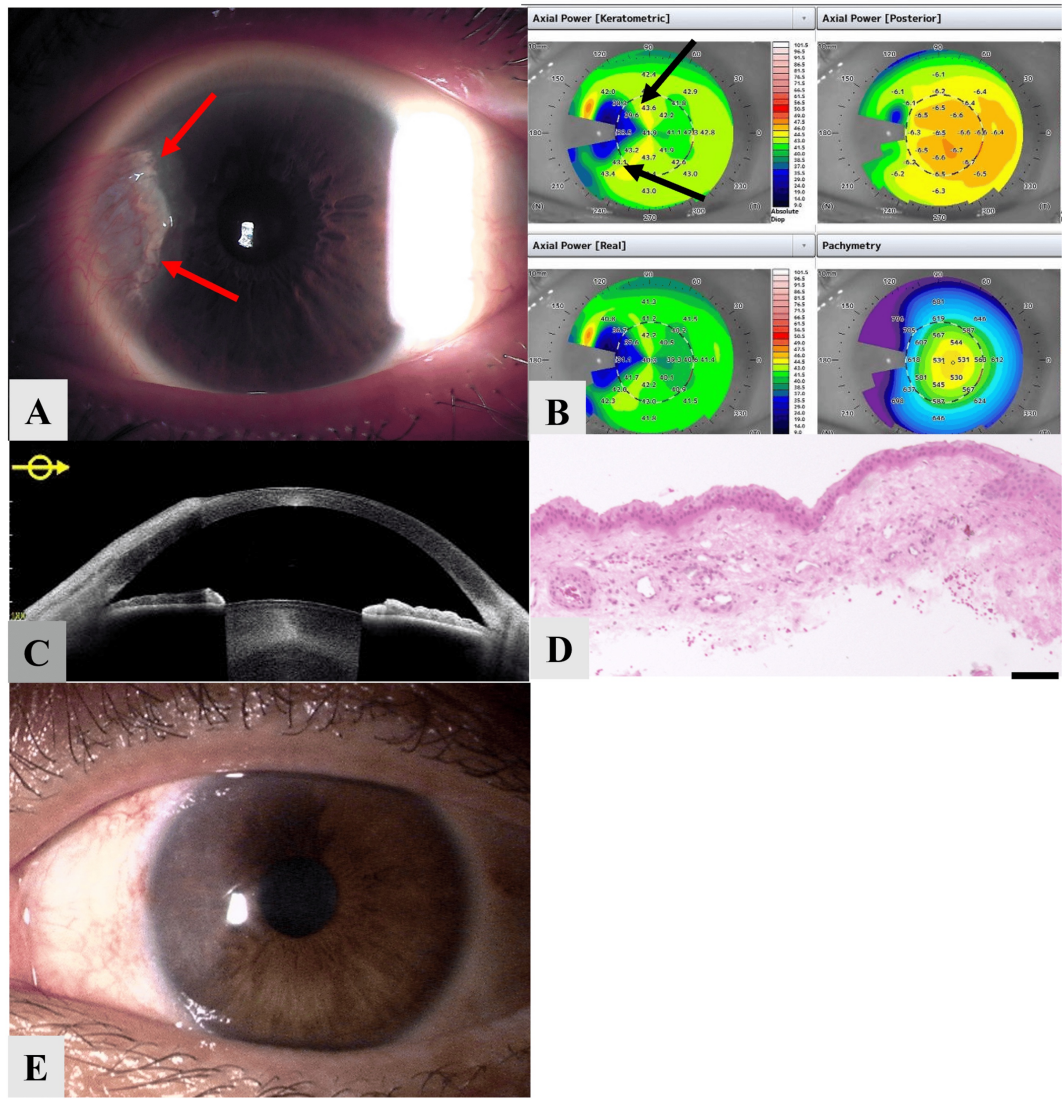
An example of pterygium in one patient following LASIK (A) A representative photograph of the anterior segment in a patient with pterygium following LASIK surgery. Red arrows indicate the presence of pterygium in the nasal area, overlying the LASIK flap. (B) Corneal topography showing irregular astigmatism in the nasal area (indicated by black arrows). (C) Anterior segment optical coherence tomography showing a superficial depth of invasion into the stroma. (D) Histological examination of the pterygium tissue showing that the corneal tissue was not identified in the section (bar=100 μm). (E) Postoperative anterior segment image demonstrating good cosmetic and functional outcomes. LASIK: laser in situ keratomileusis

An example procedure, as well as the removed tissue for histopathology study, is shown in Figure 2.

Statistical analysis

Visual acuity measurements, initially recorded as decimal values, were converted to logarithmic values (logMAR) for statistical analysis. The Wilcoxon signed-rank test was employed to compare preoperative and postoperative outcomes. All statistical analyses were conducted using the R software (version 4.2.1; R Foundation for Statistical Computing, Vienna, Austria). A p-value of <0.05 was considered statistically significant.

Results

This study enrolled four patients (three men and one woman) with a mean age of 52.8±5.1 years. The preoperative parameters are summarized in Table 1, and the postoperative outcomes are presented in Table 2. The mean follow-up period was 12.48±16.06 months. No intra- or postoperative complications were observed. The mean BCVA remained stable, with preoperative and postoperative values remaining stable at -0.15±0.04 (all values were the same). The mean uncorrected visual acuity (UCVA) was -0.15±0.04 preoperatively, and -0.04±0.09, showing no significant difference (p=0.18). Corneal astigmatism decreased from 3.33±2.54 D preoperatively to 1.08±0.32 D postoperatively (p=0.25). Conversely, anterior corneal HOA (6 mm) analysis diameter improved from 3.21±2.38 μm to 0.99±0.23 μm (p=0.125), while posterior corneal HOAs decreased from 0.22±0.11 μm to 0.12±0.02 μm (p=0.11). In Case 2, pterygium recurrence was observed 21 months after the initial surgery, necessitating a secondary surgical intervention.

**Table 1:** Demographics and clinical characteristics of the four patients before surgery F: female; M: male; OD: right eye; OS: left eye; UCVA: uncorrected visual acuity; BCVA: best-corrected visual acuity; HOAs: higher-order aberrations

Case	Sex	Age	Eyes	UCVA (logMAR)	BCVA (logMAR)	Corneal astigmatism (D)	HOAs total (6 mm)	HOAs anterior (6 mm)	HOAs posterior (6 mm)
1	F	44	OD	-0.18	-0.18	1.39	1.01	0.99	0.11
2	M	56	OD	-0.08	-0.08	4.22	3.7	3.71	0.20
3	M	55	OD	0	-0.18	0.64	1.34	1.24	0.16
4	M	56	OS	-0.08	-0.18	7.08	6.65	6.89	0.41

**Table 2:** Postoperative clinical outcomes at the final examination *In Case 2, a secondary surgery was required due to recurrence, including superficial keratoplasty and intraoperative mitomycin C application, and amniotic membrane transplantation was successful without any further deterioration. F: female; M: male; UCVA: uncorrected visual acuity; BCVA: best-corrected visual acuity; HOAs: higher-order aberrations

Case	Sex	Age	UCVA (logMAR)	BCVA (logMAR)	Corneal astigmatism (D)	HOAs total (6 mm)	HOAs anterior (6 mm)	HOAs posterior (6 mm)	Complications
1	F	44	-0.18	-0.18	0.72	0.71	0.75	0.11	None
2	M	56	-0.08	-0.08	1.60	1.15	1.14	0.10	Recurrence*
3	M	55	0.05	-0.18	1.05	0.74	0.78	0.13	None
4	M	56	0.05	-0.18	0.94	1.33	1.30	0.15	None

## Discussion

This is the first case series (n=4) to evaluate the surgical outcomes of pterygium excision in cases in which the lesion extended beneath a LASIK flap. It is valuable to indicate the safe and effective technique to treat pterygium in patients with LASIK flaps without negative impacts on UCVA as well as BCVA, because patients undergoing refractive surgeries expect better vision without glasses. Briefly, functional and refractive success should be required. Previous reports are limited, with only one case reported by Lopez and Sayegh [9], in which lesion removal was conducted using a crescent knife. Building upon their work, our study includes four cases that underwent surgery using a Maeda-type DLK spatula, which allowed for more controlled, tension-free dissection. No intra- or postoperative complications were encountered in our cohort, and the visual outcomes remained stable.

Although one patient experienced recurrence requiring a secondary procedure involving amniotic membrane transplantation, all cases showed significant improvements in parameters, including corneal astigmatism and HOAs. Notably, corneal astigmatism decreased from 3.33±2.54 D preoperatively to 1.08±0.32 D postoperatively. Furthermore, both anterior and posterior corneal HOAs tended to improve after surgery. BCVA remained stable throughout the perioperative period, likely because of the high baseline visual acuity of the study population.

The strength of this study lies in the successful preservation of the LASIK flap during pterygium excision. Because LASIK flaps lack sutural fixation, they are vulnerable to displacement due to external mechanical forces, such as ocular trauma or surgical manipulation. We implemented multiple intraoperative strategies to minimize flap stress and reduce this risk. These included performing a tension-free dissection from the lesion head toward its body using a DLK spatula, avoiding upward traction during tissue handling, and employing intraoperative fluorescein staining to precisely delineate the depth of resection and optimize stromal smoothness. Fluorescein staining further enabled us to assess the flap integrity in real-time, allowing us to immediately detect any damage to the LASIK flap. Notably, the use of a Maeda-type DLK spatula allowed for gentle and controlled tissue dissection. Its blunt, curved design allows lamellar separation with minimal mechanical stress. This makes it particularly well-suited for tension-free excision near the LASIK flap.

The findings of the present study highlight the importance of implementing precise surgical techniques and careful preoperative planning, given the surgical challenges associated with pterygia extending beneath LASIK flaps. Even in the case of recurrence (Case 2 herein), the recurrent lesion was excised without compromising the structural integrity of the LASIK flap. The postoperative course was stable after secondary surgical intervention, and there was no evidence of recurrence during the 12-month follow-up period. Although pterygium can break Bowman's layer structure [11], in our cases, the stromal part of the LASIK flap was preserved without any injury because the histological examination of pterygium tissue did not include any corneal tissue identified in the sections.

The drawback of this study was the existence of recurrence in Case 2. In Case 2, the patient did not adhere to the prescribed postoperative regimen, including topical steroids, antibiotics, and tranilast, nor did they attend regular follow-up visits. The recurrence was likely associated with this lack of adherence, and the patient was referred back to our hospital after visual deterioration because a recurrent pterygium became evident. Pterygium recurrence is primarily attributed to the proliferation of residual fibrovascular tissue, which is exacerbated by postoperative inflammation, angiogenesis, and excessive ultraviolet exposure. Disruption of the limbal stem cell barrier plays a central role in the conjunctival overgrowth of the cornea. The surgical technique is also critical; the bare sclera method carries the highest recurrence risk, whereas conjunctival autografting significantly reduces recurrence compared to amniotic membrane transplantation. Younger age and inadequate control of postoperative inflammation further increase the likelihood of recurrence.

Limitations

This study has several limitations, primarily its retrospective nature and the small number of cases, which reflects the rarity of this complication. The small number of cases resulted in a lack of statistically significant differences in the evaluated factors. Further studies with longer post-LASIK follow-up after LASIK are warranted.

## Conclusions

Pterygium excision with pedicle conjunctival flap transplantation was successfully performed in all four cases involving pterygium invasion of the LASIK corneal flap. Meticulous surgical techniques, including careful lesion excision and intraoperative assessment of resection depth, are crucial in preventing flap displacement and associated complications. Favorable outcomes were achieved with secondary transplantation, including superficial keratoplasty, intraoperative mitomycin C application, and amniotic membrane transplantation, even in cases where recurrence occurred. These results suggest that, with careful surgical planning, pterygium involving the LASIK flap can be treated safely while preserving both functional vision and cosmetic appearance.
